# The Figure of the Staggering Rat: Reading Colonial Outbreak Narratives Against the Grain of “Virus Hunting”

**DOI:** 10.1093/jhmas/jrae004

**Published:** 2024-05-03

**Authors:** Christos Lynteris

**Affiliations:** University of St Andrews, Scotland, UK

**Keywords:** plague, rats, epistemology, cosmology, colonialism, China, zoonosis

## Abstract

The image of dazed, plague-infected rats coming out of their nests and performing a pirouette in front of the surprised eyes of humans before dying is one well-known to us through Albert Camus’s *The Plague* (1947). This article examines the historical roots of this image and its emergence in French missionary narratives about plague outbreaks in the Chinese province of Yunnan in the 1870s on the eve of the Third Plague Pandemic. Showing that accounts of the “staggering rat” were not meant as naturalist observations of a zoonotic disease, as is generally assumed by historians, but as a cosmological, end-of-the-world narrative with a colonial agenda, the article argues for an approach to historical accounts of epidemics that does not succumb to the current trend of “virus hunting” in the archive, but rather takes colonial outbreak narratives ethnographically seriously.

Rats, in one form or another, can be found depicted in the vast majority of book covers of what is without a doubt the most influential modern literary work on an epidemic, Albert Camus’s *The Plague (La peste*), originally published in 1947.^1^ Even those who have not found an opportunity to reread the book during the COVID-19 pandemic may recall one of the most enduring images of Camus’s allegory for fascism, told as the story of an epidemic of bubonic plague in colonial Oran: rats coming out of their hiding places, dazed and confused, performing a strange swirl around themselves and dying before the astonished eyes of the novel’s human protagonists. In a manner unusual for a work of literature, the figure of the staggering rat appears twice in the memorable chapter two of the book:

That evening, when Dr. Rieux was standing in the entrance, feeling for the latch-key in his pocket before starting up the stairs to his flat, he saw a big rat coming towards him from the dark end of the passage. It moved uncertainly, and its fur was sopping wet. The animal stopped and seemed to be trying to get its balance, moved forward again towards the doctor, halted again, then spun around itself with a little squeal and fell on its side. Its mouth was slightly open and blood was spurting from it.^2^

Then a few pages later, rats, apparently infected with plague, are once again seen behaving in an out-of-place and out-of-character manner, only this time the narrative generalizes from a single rat to the population of the animals in the Algerian city: “From basements, cellars and sewers they emerged in long unwavering files into the light of the day, swaying helplessly, then did a sort of pirouette and fell dead at the feet of horrified onlookers.”^3^

What is the source of this curiously repetitive, almost mimetic image, whose examination has escaped the scrutiny of both scholarly works on Camus’s novel (including its treatment of animals) and the numerous commentaries on *The Plague* arising during the COVID-19 pandemic?^4^ Surprisingly perhaps, the origins of Camus’s narrative lie in an epidemic of bubonic plague that struck Yunnan, a province of the Chinese Empire, in the 1860s-1870s, an outbreak widely recognized by historians as the origins of the devastating Third Plague Pandemic, which within three decades of its first major outbreak in Hong Kong (1894), spread to all inhabited continents and led to more than twelve millions deaths across the globe.^5^ My interest here is not to show the influence of epidemiological narratives on Camus. Rather, taking Camus’s passages as a point of what Walter Benjamin calls “profane illumination,” in this article I examine the emergence of the figure of the staggering rat within its concrete historical-ethnographic context.^6^ Through this investigation, I argue for an approach contrary to the prevailing historiography of the Yunnan plague, which has used available sources in order to establish a retrospective diagnosis of the epidemic and its zoonotic origins.^7^ Instead, I argue that the figure of the staggering rat needs to be taken ethnographically seriously and examined within the ontological and political worldviews and agendas of those articulating it and employing it in late nineteenth-century outbreak narratives.^8^

Beyond its immediate value in relation to the historical study of plague, or the history of medicine in late-Qing China, this reading of the figure of the staggering rat offers a methodology for rethinking and re-approaching epidemic and pandemic histories. For the critical examination of the figure of the staggering rat holds a stark warning for historians and anthropologists engaged in the study of epidemics: reading archival and ethnographic sources that contain outbreak narratives *with the grain* of contemporary or hegemonic concerns and preoccupations, such as zoonotic emergence, runs the very serious risk of misreading the historical and ethnographic reality in question. It does so by acting in a “virus hunting” manner, which mines, decontextualizes, and reduces available sources (texts, stories, memories, myths, rituals, daily practices, material culture) into “epidemiological evidence” regardless of the intentionality of the informants (for lack of a better word) in question or the meanings, actions, and complex social and interspecies relations involved in and afforded by the said discourses or practices in the context of their articulation or performance.^9^ The danger here is double. When on the one hand, the discourse or performance is articulated or undertaken by indigenous, subaltern, or colonized subjects, the virus-hunting approach contributes to what I have elsewhere described as the epistemological enclosure of diverse systems of knowledge and lifeways.^10^ This is a process that dispossesses the latter of their epistemological and ontological autonomy in order to render them commensurable with hegemonic systems of knowing and being. When,on the other hand, as in the case examined in this article, the discourse or performance in question is articulated or undertaken by colonial, state, corporate, or otherwise hegemonic subjects, the virus hunting approach risks blinding us to the power and knowledge agendas of these agents, and the ways in which facts or evidence are configured in order to foster and legitimize processes of exploitation and domination.

In this way, our justified concerns over currently pressing questions, such as the emergence of zoonotic diseases or the origins of epidemics during the ongoing COVID-19 pandemic, risk becoming what Eduardo Viveiros de Castro has called “epistemological tranquillizers.”^11^ In other words, they disallow us to understand our historical or ethnographic informants’ entanglement with the non-human world, their own understanding of disease, or indeed the use of outbreak narratives in order to talk about more-than-disease related realities and bring about more-than-disease related impacts on the world. This article is written against the background of four years of such virus-hunting practices in the course of the COVID-19 pandemic.^12^ It is a call against this resurgent, neocolonial grain of approaching outbreak narratives, and for taking these narratives ethnographically seriously. Far from being an anthropological cliché, this approach allows us to maintain the epistemological and ontological autonomy of indigenous and subaltern lifeways while at the same time fostering our understanding and deepening our critique of the ways in which outbreak narratives have been used to further imperial, state, corporate, and other hegemonic worldviews, interests, and agendas.

## The Yunnan Plague

Plague outbreaks in Yunnan during the nineteenth century are accepted today as the source of the Third Plague Pandemic, with narratives of ratfalls (rat epizootics) in mid-nineteenth-century sources being treated as indexes of actual rat epizootics preceding outbreaks of plague among humans.^13^ At the same time, extensive research on plague in the region has shown that Yunnan continues to maintain significant sylvatic reservoirs of the disease.^14^ Carol Benedict convincingly demonstrated that Chinese records point at the potential existence of plague in Southwest China as early as the late eighteenth century, with contemporary Chinese commentary on ratfalls more recently examined in detail by Wilt L. Idema.^15^ However, there is no indication that the authors initiating and developing narratives about ratfalls preceding human plague (all of them French or British) in 1870s Yunnan were aware of these sources. It is more probable that at least the medical authors involved in this process (Patrick Manson in particular) may have encountered earlier narratives of ratfalls in association with plague developed by British colonial doctors in the Indian Himalayas.^16^ No direct evidence of this exists, however, in the sources examined in this article, and in consistence with prevailing histories of European framings of the rat, the outbreak narrative emerging in Yunnan verifies that the rat-plague association was novel to Europeans at the time.^17^

Plague was mentioned alongside other disasters in the region in missionary documents from Yunnan as early as 1866.^18^ Beginning in the early 1870s, missionary reports and letters began associating outbreaks of a devastating epidemic in Yunnan with the end of the Panthay Rebellion — an Islamic insurrection between 1856 and 1873 that challenged Qing sovereignty in the South-West province of the Empire, leading to the establishment of a short-lived Sultanate.^19^ The death toll of the rebellion was enormous (about two million), and missionaries, as well as explorers, wove together an image of war- and epidemic-driven ruination in the province, with the discussion of the epidemic being generally used as an appendix of or support for narratives around the ravages of the Panthay Rebellion, especially as these affected local communities that had converted to Christianity. The first extensive record of a discussion of the disease is found in this context, in the missionary correspondence of the Missions Étrangères de Paris (MEP), which maintained a very active presence in the region since the 1850s, and which was greatly affected by and preoccupied with the Islamic insurrection.^20^

On 27 July 1874, Joseph Ponsot (1803-1880), Bishop of Philomélie for the Apostolic Vicariate of Yunnan, informed the members of the central council of the Society of the Propagation of the Faith (Œuvre pour la Propagation de la Foi) – a Pontifical Mission Society founded in Lyon in 1822, which supported Catholic missions across the world – that following the end of the hostilities, a devastating epidemic struck the province. This appears to be the first document in a European language discussing at length what came to be known as the Yunnan plague. Strikingly, however, the report did not associate the epidemic with bubonic plague. Instead, Ponsot stressed that this was “an epidemic unknown in Europe.”^21^ The only mention of “plague” in Ponsot’s letter was a generic one, where *la peste* was used in the sense of an epidemic or pestilence rather than that of bubonic plague, as understood at the time. Ponsot wrote that the “terrible epidemic” (*terrible peste*) called “Cang-tsy-tchén” was in fact first noted by another MEP missionary, Jean Marie Le Guilcher:

The people, he wrote, call it the disease of rats. The rats start by being affected by an epidemic, dying in heaps. I saw one of my catechists remove from his old hovel two bushels of rotten rats. The infection is strong, and it is it that undoubtedly that gives the contagion to man in the plains of Tsou-hiong-fou. There are villages where the disease returns every year. As soon as the rats die, the inhabitants emigrate to the mountains, where they wait two or three months, and once the bad odour has disappeared from their home, they can return there with impunity. It should be noted that the mountains are much more salubrious than the valleys. From the beginning of August last year, the Minjia father, Paul Tchao has occasion to visit me. He told me that rats died in immense quantities in Pien-kio (his district). We did not pay much attention to it at first, as the disease had never appeared in this country.^22^

Jean Marie Le Guilcher (1828-1907) had been part of the MEP’s Yunnan mission since 1853, and since 1868 had been leading the mission in Ta-pin-tse. The source of Ponsot’s quote is obscure as no surviving letters or other documents by Le Guilcher mention the Yunnan epidemic. However, Ponsot’s letter was soon to enjoy wide circulation as it was published in the 1875 volume of the *Annales de la Propagation de la Foi*, the main publication of the Society for the Propagation of the Faith, where it was published with several changes.^23^ Significantly, where the two versions differed most extensively was their account of indigenous responses to ratfalls. The manuscript and published versions of Ponsot’s rendition of Le Guilcher’s account of the Yunnan epidemic differed in the testimony about villagers fleeing to the mountains. The manuscript contained an etiological narrative that identified the cause of the epidemic as miasmatic (odors) and which endorsed the choice of the indigenous population to flee to the mountains (where the air was supposedly free from miasma) until the odour had disappeared from the villages; the published version of the letter made no mention of this. In the manuscript it is clear that, according to Le Guilcher, locals fled not ratfalls or a disease spread by rats, but the miasma that killed the former. By contrast, a key passage in the publication, “The infection, which they spread, is undoubtedly the case of the contagion which attacks man in the plains of Tsou-hiong-fou,” unambiguously stated that rats did not simply suffer from but also spread the disease; this passage was not, however, part of the manuscript.^24^

Who edited Ponsot’s letter and to what purpose is not known, as no archives survive on the matter. What is clear is that in its published form, which soon came to be read by future authorities on the disease, the miasmatic etiology of the original letter was transformed into a “contagious” one, involving the transmission of the disease from rats to humans. Given the hotly debated question of plague’s infectiousness and contagiousness in the course of the nineteenth century, this did not deny the miasmatic origin of the disease, but added a new trait to it in terms of its transmission. It thus transformed the disease’s ontology and introduced for the first time the idea of rat-to-human plague infection.^25^

## Rats, Missionaries, and Gunrunners

In the following years, the epidemic in the region continued to be discussed in French missionary letters and publications.^26^ Yet it was another, unexpected source that elicited international medical interest in the Yunnan plague. This was the report of a British-directed murder investigation/punitive expedition in 1876 led by Thomas Grosvenor following the murder of Augustus Raymond Margary, a British consular official in Yunnan; an “affair” with important political consequences for British-Chinese relations.^27^ The account of the investigation/expedition, written by Edward Colborne Baber, contained information gathered by another key MEP missionary in Yunnan and important player in the “Margary affair,” Jean-Joseph Fenouil (1821-1907).^28^ In Baber’s report, Fenouil was quoted as claiming that the plague that had struck Yunnan in previous years affected not only humans but also domestic animals and rats. This was the first mention of the figure of the staggering rat:

[Plague’s] approach may often be known from the extraordinary movements of the rats, who leave their holes and crevices, and issue on to the floors without a trace of their accustomed timidity, springing continually upwards from their hind legs as if they were trying to jump out of something. The rats fall dead, and then comes the turn of the poultry; after the poultry have succumbed, pigs, goats, ponies, and oxen successively die off.^29^

By contrast to Ponsot’s published letter, in Fenouil’s account, there was no indication that rats or other animals transmitted the disease to one another or to humans. Instead, it was stated that the epidemic was caused by a “pestilential emanation slowly rising in an equable stratum from the ground, and as it increases in depth, all animals are as it were drowned in its poisonous flood; the smaller creatures being first engulfed, and man, the tallest of Yunnan animals, suffering last.”^30^

Baber’s report of Fenouil’s testimony, published in 1878, was strikingly similar to another account of plague in Yunnan published the same year by no less than Patrick Manson, who in the decades that followed became the most celebrated so-called father of tropical medicine. In his *Report on the Health of Amoy for the Half-year ended 31*^*st*^*March 1878* for the Chinese-controlled but internationally-led Imperial Maritime Customs Service (IMCS), Manson translated and reproduced notes on the Yunnan plague authored by Émile Rocher, adventurer and future French diplomat to China, who had taken part in a mission to the region in 1871-1873.^31^ In “Notes on the Plague in Yunnan,” Rocher related that the disease was locally called *Yang-tzŭ,* which historian Marta Hanson has explained as “meaning severe itching of the skin or pruritus,” but which was, in the context of colonial writings on plague in the region, understood as relating to “boils.”^32^ Following prevalent etiologies of plague at the time, Rocher saw the epidemic as caused by telluric gases: “There is a fact that inclines one to think that the epidemic is owing to exhalations from the soil and it is this, those animals that live in the ground, in drains or in holes, are the first to be attacked. This is particularly noticeable with the rats.”^33^ In Manson’s translation, Rocher then proceeded to give an evocative account of ratfalls that, carrying as it did the figure of the staggering rat in an amplified form – with rats appearing in militarised form as “troops” – would prove to have an important impact on framing rat epizootics in relation to plague outbreaks for decades to come: “As soon as these animals are ill, they leave their holes in troops, and after staggering about and falling over each other, drop down dead. The same phenomenon occurs in the case of other animals, such as buffaloes, oxen, sheep, deer, pigs and dogs. All are attacked, but the dog less severely than the others.”^34^

Based in Amoy (Xiamen) at the time, Manson had no direct experience of the epidemic and did not contribute any information in relation to the plague in Yunnan. However, his translation differed on several points from Rocher’s French text, which was published in 1879-1880 as an appendix to the two-volume book titled *La Province Chinoise du Yün-nan*. Where Manson’s translation read “As soon as these animals are ill, they leave their holes in troops, and after staggering about and falling over each other, drop down dead,” Rocher’s text was even more striking in its imagery: “Once they fall ill, they come out in groups [bandes], burst into the interior of the houses, run in a panic, and after a few turns on themselves, fall dead.”^35^

As Jean Michaud has shown in his history of French Catholic missions in the region, what historians usually describe as a “diplomatic” mission led by the explorer Jean Dupuis (to which Rocher was attached) was in reality a gunrunning expedition “launched in the wake of the recent agreement made with the Yunnan authorities aimed at providing local rulers with the Western weaponry they needed to crush the Muslim uprising.”^36^ Rocher should be seen as a quintessential colonial agent, as he found the time to combine gunrunning with geoprospecting, “investigating the mineral resources of the region.”^37^ Rocher’s book, Michaud argues, was “designed for administrative consumption, with the aim of informing on the topography, the layout of roads, the communication systems, the regional history, the current rebellions, production and commence, and, in particular, indigenous metallurgy.”^38^ Rocher’s notes on plague should thus be scrutinised as part of this colonial project: as information collected and relayed not for the benefit of detached scientific knowledge, but rather for immediate imperialist exploitation.

Historians and life scientists writing on the Yunnan plague *with the grain* of virus hunting have overlooked the aim and context of the information collected by Rocher, taking his account at face value. They have not submitted it to the scrutiny one would usually reserve for a report collected in the process of running guns and whose aim was to facilitate the colonial take-over, financial or indeed military, of the region described.^39^ At the same time, also overlooked in virus-hunting histories of the Yunnan plague are the different evidentiary styles in Rocher’s account. Maintaining a critical distance from the information related in Rocher’s account of plague as a product of French imperialism is as important as taking ethnographically seriously its tropes and tones of witnessing.

Rocher’s book is composed by diary-like accounts, general descriptions of the expedition in non-diary form, as well as analytical passages on the history of the region, the Panthay Rebellion, and the Yunnan plague. Different discussions of plague in the book, both in its main body and in the “Notes on the Plague in Yunnan” appendix, reflect these narrative differences and their evidentiary claims. The first mention of plague in Rocher’s book (volume 1) was in his entry for 26 January 1871, where he noted that the expedition entered a rice-cultivation area whose inhabitants “had fled before the plague, which over several years, has made appalling ravages in the country.”^40^ It is in this passage that rats also made their first appearance in Rocher’s narrative:

This illness, which is no other we think than a plague in the form of the buttons of Aleppo, breaks out most frequently in the month of May and continues to strike until the month of November, but changing location. What makes us think that this epidemic is due to soil exhalations is that rats are the first victims of the scourge; once they are ill, they come out of their holes, take refuge inside habitations, make a few turns on themselves and die. Buffaloes, oxen, sheep and goats are then struck, but among these the evil has a lesser hold.^41^

While, as a result of adopting a virus-hunting approach, medical authors and historians alike have assumed Rocher to be providing an eyewitness account of ratfalls, no such claim is made in the passage quoted above, major parts of which (on soil exhalations and rats leaving their nests and making a gyration before dying in the open) were repeated in his “Notes on the Plague in Yunnan” (appended to the second volume of the book). Could Rocher be simply reproducing Fenouil’s discourse, either through conversations with him or by exposure to Baber’s published account, without making mention of him as his source?^42^ This possibility is reinforced by the fact that the way evidence was presented in the passage differed from the way in which Rocher marked eyewitness accounts elsewhere in his narrative. Let us take the example of a paragraph where he described the expedition’s impressions from Li-chia-ying. This is the second passage in the book where ratfalls were mentioned. Here Rocher’s description of the devastation employed two testimonial registers: an eyewitness account, followed by a statement of a generally known fact (which I highlight here in italics), followed by an eyewitness account:

At the moment of our passage, the village was entirely deserted, the inhabitants having fled their abodes in order to camp on the heights, abandoning their growing crops, so as to escape the attack of an enemy more ruthless than the rebels, the plague. *That year, the terrible scourge had stricken with such furry on the unlucky district, that almost all horned beasts, following the rats, perished*. So it was only with great difficulty that we managed to find an old temple to spend the night there, and our servants were forced to go to the heights to stock up on the essentials. This small place had the aspect of a desert: no one in the fields or in the houses, wreaths of rice lay in places, half made and as if someone would come to finish them. No domestic animals, no more smoke rising from the chimneys, no noise, no movement; life seemed suspended.^43^

Whereas the abandonment of the village at the beginning and end of the paragraph was accounted as a direct, first-hand experience, by contrast the death of rats and other animals from plague was simply reported as a generally known fact. If Rocher encountered dead rats or ratfalls on 26 January, he never made such a claim. Indeed the entry on that date clearly stated that the disease strikes “in the month of May and continues to strike until the month of November.” Being made in late January, his account was thus one witnessing the aftermath of the epidemic, not its course, as is often assumed by historians, with information about phenomena such as ratfalls and other epizootics being second-hand and not the result of direct observation.

As plague spread from Yunnan to other parts of southwest and southern China, Rocher’s account of plague played an important role in fostering the figure of the staggering rat as a *sine qua non* of outbreak narratives about plague in China. In some cases, this involved no more than directly reproducing (often without acknowledgement) Rocher’s narrative to describe outbreaks in other regions of the country.^44^ In others, it involved weaving together more information about ratfalls, often entangled with plague etiologies involving human corpses and contaminated soil.^45^ From there, the figure of the staggering rat entered the realm of medical literature engaged with the Third Plague Pandemic, as this proceeded from Hong Kong in 1894 to spread across the globe. By the start of the twentieth century, accounts of plague in different parts of the world would rehearse the figure of the staggering rat in various permutations, so much so that we can say that this figure became both a light motif of plague narratives and an index of authenticity in observations about ratfalls preceding human epidemics.

Following this genealogy, and keeping the evidentiary ecology of the development of outbreak narratives involving the staggering rat in mind, it should be clear that approaching accounts of ratfalls in the context of the Yunnan plague in the 1870s as a source of epidemiological evidence, in other words in a virus-hunting manner, is enormously problematic. This is not so much because we do not have a single unambiguously first-hand account of this phenomenon, as due to the epistemological violence and obfuscation that are involved in reducing the outbreak narratives in question to repositories of data about the zoonotic aspects of plague in the region. Instead of poaching in the archive for evidence of zoonosis, what is needed here in order to understand the affordances of the figure of the staggering rat is to situate it within its immediate ontological and political environment. In other words, we need to take the figure of the staggering rat ethnographically seriously. This in turn offers broader methodological guidance as regards to epidemic/pandemic histories, if our aim is to understand the emergence of “scientific facts” in their epistemic, social, and political complexity, and not simply to project our own framings or configurations of epidemic/pandemic crises to the past.

## The Great Chain of Infection and the Staggering Rat

Reading the various iterations of the figure of the staggering rat in the 1870s Yunnan literature, it becomes immediately clear that this is embedded in a much broader outbreak narrative regarding the death of non-human animals as a result of the epidemic. We need to examine this narrative as it forms the ontological and political ground of the figure that interests us. This outbreak narrative concerns what we may call the “great chain of infection.” This is a story absent from the Ponsot/Le Guilcher account, and first appearing in Baber’s account, where Fenouil states the death of poultry following that of rats, itself being followed by the successive death of “pigs, goats, ponies, and oxen” – a chain of infection attributed to “pestilential emanation slowly rising in an equable stratum from the ground,” engulfing different creatures in succession of height.^46^ In Rocher’s narrative, by contrast, we do not find a chain of infection determined by the height of each animal, but simply a collection of susceptible beasts. In one passage these are said to include “buffaloes, oxen, sheep, deer, pigs and dogs,” while in another passage only “Buffaloes, oxen, sheep and goats.”^47^

We can thus say that accounts of the Yunnan plague contain two mythic variants linking the disease to animals. The weak variant, seen in Rocher, simply presented animals as dying at the same time as humans, stricken with plague in no particular sequence or order. This is an iteration of a well-known classical literary trope, which first appeared in Latin descriptions of *pestis*– a term not reserved for plague, as this came to be known from the sixth century AD, but more generally to epidemics. The image of an epidemic striking both humans and animals appeared in Vergil’s *Georgics III*, in the famous description of the Noric plague, which killed calves, pigs, and horses. So miasmatic was the atmosphere of Noricum said to be, that it caused even birds to fall dead in mid-flight: “Here once arose a season pitiable for the diseased atmosphere, and through-out all the seething of autumn it glowed white hot and gave over every kind of beast to death, and every [race of] wild animals.”^48^ Hunter Gardner has shown how Vergil’s narrative on the Noric plague relied on Lucretius’s earlier configuration of the so-called plague of Athens (again not plague in biological terms) in terms of civil war as well as, on a more ontological level, a “process of decay and the collapse of boundaries within *corpora*.”^49^ The disease striking across the human/non-human ontological barrier marked not simply an all-engulfing epidemic, but one that threatened the order of things with *discordia*.^50^

To find this onto-political narrative reflected in Rocher’s account of plague in Yunnan is not surprising as it had been reproduced by the physician and historian of medicine Justus Hecker in his 1832 book on the Black Death (*Der schwarze Tod im vierzehnten Jahrhundert*), an immensely popular and widely translated work that has been shown to have introduced a new “historical and nosological” framework of plague, which transformed understandings of the epidemics of 1347-1351 and invested plague with catastrophic, world-historical consequences.^51^ Hecker argued that during the Black Death, “multitudes of dogs, cats, fowls and other animals, fell victims to the contagion; and it is to be presumed that other epizootes among animals likewise took place, although the ignorant writers of the fourteenth century are silent on this point.”^52^ Rather than, however, simply reproducing the Latin trope of the great chain of infection, Hecker integrated it into what Feye Marie Getz has shown to be his decisive turn to “gothic epidemiology.”^53^ This approach was unique and unprecedented in that it attributed world-historical properties of ontological proportions to an epidemic. For Hecker, the Black Death marked not simply a catastrophic event, but also one that revealed humanity’s true relation to Nature. As Getz argues, for Hecker, “The Black Death was a demonstration of the power and glory of Nature, so overwhelming in its universality and its terror as to defeat the best efforts of mere science to define it.”^54^ The way the great chain of infection functioned as a mytheme in this ontological scheme was by underlining, on the one hand, the all-encompassing power of plague over living beings, and, on the other hand, the power of Nature to abolish any distinction between humans and non-human animals through plague as an ecumenical equalizer.

The second, stronger, mythic variant found in Yunnan plague narratives involved not simply the concurrent infection and death of humans and animals, but what we can properly call “a great chain of infection”: the gradual and sequential infection of “lower” animals (exemplified in rats), cattle, and then humans. We have already seen how Baber’s account of Fenouil’s testimony formed the prototype of this mytheme as regarded the Yunnan plague. However, this was not something invented by Baber or Fenouil, but a transformation on the classical variant discussed above. The most developed articulation of this is to be found in Ovid’s *Metamorphoses* and in particular in his narrative about the plague of Aegina: “At first, in a heap of dogs and birds and sheep and oxen and among the wild beasts, the power of the sudden disease took hold.”^55^ Here what we today call a panzootic is configured as taking place in advance of the human plague, borrowing from the dramaturgical trope used by Homer in the *Iliad* (1.50-51) and by Sophocles in his narrative on the plague of Thebes in *Oedipus Rex* (25-30).

Where the two variants significantly differ in the Yunnan plague narrative is their emphasis on plague as a disease of disorder. The weak variant emphasises the manner in which plague collapses ontological barriers and, being a disease of decay, fosters a discordian state. The employment of the weak variant thus reiterates the ontological catastrophe that is supposedly inherent in epidemics. At the same time, it allows for the ecumenical ravages of plague to rhyme with those of the Panthay Rebellion, creating an onto-political metonymy of ruination, disaster and decay. The strong variant too relies on the same metonymic operation, but with a twist. Here Nature itself provides a glimpse of order within the prevailing disorder. Animals and humans do not get infected and die helter-skelter, but in a hierarchical manner, according to their height, which also happens to be, roughly speaking, a hierarchy on the scale of animality: rats, poultry, pigs, goats, ponies, oxen, and finally humans. Therefore, the strong variant provides a kind of hope, a glimpse of the reordering capacity of Nature led by “man” (White, male, bourgeois and Christian, following colonial thinking), while it contains the potential of the terrible catastrophe being used for highlighting divine providence, as the sequential death of animals could be argued to be a portent of the coming plague.^56^

It is not surprising that historians and life scientists writing on the Yunnan plague have overlooked all traces of the “great chain of infection” narrative. The fact that none of the animals mentioned therein (pigs, goats, ponies, sheep and oxen) are actually susceptible to *Yersinia pestis* casts serious doubt on virus-hunting approaches to the sources. It also challenges, from the positivist perspective of these approaches, the evidential value of accounts of ratfalls, as these are made by the same people reporting on the great chain of infection, more often than not in a continuous narrative to the latter. Our aim here should not be to correct virus-hunting approaches by trying to separate what was really observed from what was imagined. Instead, if we are to take these outbreak narratives ethnographically seriously, we need to understand the emergence of the staggering rat not as a naturalist observation opposed to the cultural relic of the great chain of infection, but rather as a figure situated and assuming agency within the latter and its ontological and political affordances.

Whereas in Ponsot/Le Guilcher we only read about rats “dying in heaps” or “en mass,” in Baber/Fenouil a new image emerged, describing how rats “leave their holes and crevices, and issue on to the floors without a trace of their accustomed timidity, springing continually upwards from their hind legs as if they were trying to jump out of something.” The image was repeated in Rocher who in Manson’s version wrote, “As soon as these animals are ill, they leave their holes in troops, and after staggering about and falling over each other, drop down dead,” while in the original the description was even more evocative: “Once they fall ill, they come out in groups, burst into the interior of the houses, run in a panic, and after a few turns on themselves, fall dead” and “once they are ill, they come out of their holes, take refuge inside habitations, make a few turns on themselves and die.” This proved to be a very popular image, with publications following Baber/Fenouil and Manson/Rocher not failing to further embroider it. In an 1886 article on the Yunnan plague published in *Les missions catholiques*, for example, we read an intricate weaving together of Baber/Fenouil’s “timidity” and Manson/Rocher’s “staggering” images: “Forgetting their natural shyness, [the rats] come out of their hole in broad daylight, and run hither and tither with no fixed purpose (*courir çà et là à l’aventure*). They make a few steps in painful trotting, then they stop, breathless, and they soon end up succumbing.”^57^

The source of this evocative image has in fact nothing to do with rats and is no other than the well-known passage from Boccaccio’s *Decameron*, given here in John Payne’s translation:

Of this mine own eyes (as hath a little before been said) had one day, among others, experience on this wise; to wit, that the rags of a poor man, who had died of the plague, being cast out into the public way, two hogs came up to them and having first, after their wont, rooted amain among them with their snouts, took them in their mouths and tossed them about their jaws; then, in a little while, after turning round and round, they both, as if they had taken poison, fell down dead upon the rags with which they had in an ill hour intermeddled.^58^

The Yunnan plague narrative borrowed the image of the swirling hogs and transposed it onto rats. This image would have been readily available to authors at the time both directly from *Decameron* and from Hecker’s mention of the scene as evidence of the epizootic nature of plague: “Thus Boccaccio himself saw two hogs on the rags of a person who had died of plague, after staggering about for a short time, fall down dead, as if they had taken poison.”^59^ However, in transposing the image of the staggering hogs onto rats, the Yunnan plague narrative added an important ontological layer that may be easily neglected were we to ignore the intertextual nature of outbreak narratives. For the image of the rat abandoning its natural ways, coming out in the open, and swirling around itself for all to see before dying is consistent with a particularly Latin configuration of *pestis*. This, as Gardener has shown, depicted affected animals as transgressing the boundaries of their nature (a metaphor of civil strife in Vergil).^60^ David West has evocatively described this image as mobilizing anthropomorphism so as to deliver a powerful poetics of “pathos and paradox.”^61^ Gardner explains how this transformation/transgression is brought about by means of “the internal conflicts that riddle the bodies of plague victims gradually play[ing] themselves out”: “As birds and sea creatures abandon their natural habitats (541–3; 546–7), predatory animals (wolves and dogs, 537, 540) no longer stalk their accustomed prey (sheep and deer, 537, 539). An eerie peace ensues, though it is a tranquillity achieved through death rather than concord.”^62^

The figure of the staggering rat in 1870s outbreak narratives about plague in Yunnan may thus be said to be emblematic of the ontological and political affordances of the great chain of infection mytheme within it was embedded. A “vermin” increasingly invested in the course of the nineteenth century with gothic meanings and agency, the rat was an ideal protagonist in an outbreak narrative that, on the one hand, incorporated the onto-politics of “gothic epidemiology,” while on the other hand, was about much more than simply a disease.^63^ Both in missionary accounts and in Rocher’s narrative, the staggering rat embodied the process of *panolethria* (from the Greek *pan* for all or all-encompassing, and *olethros* for disaster) imagined to be taking place in Yunnan at the time, “a nexus of calamities” where all disasters seem to be working “in tandem” in a manner “involving simultaneous political, climatological, seismological, and pandemic crises” – or in the case of Yunnan, famine, the Panthay Rebellion, and plague.^64^ Far from being simply symbolic, through its emblematic behaviour of abandoning its true nature and of revealing this metonymic ontological collapse to humans in a most dramatic manner, the figure of the staggering rat played a key part in a catastrophic experience of the world that advocated conversion and colonization as the interlinked solutions to what its authors saw as a double, indigenous and natural, challenge to Empire's and God’s order.

## Conclusion

The figure of the staggering rat in the Yunnan plague outbreak narrative must be recognized as aimed not at describing an observed fact, but rather at conjuring up an image of plague as a disease that transforms and transgresses the nature of things. It was a figure associated to a generalized state of *discordia*, which could in turn be redeemed, in the eyes of the people articulating this outbreak narrative, through colonization and conversion. Rather than engaging in virus hunting for zoonosis in the writings of missionaries, gunrunners, and members of punitive expeditions in 1870s Yunnan in an effort to historically validate current passions for identifying epidemic origins, we need to take their worldview ethnographically seriously. This is perhaps professionally more difficult than when this need arises in relation to indigenous, colonized subjects with whom we tend to sympathize as anthropologists or historians. However, this approach is the only one that can unburden us from the presentist bias of virus hunting and help us understand how the figure of the staggering rat was part of a colonial, onto-political vision of the world in a particular moment and place when imperial power was seriously contested.

COVID-19 has provided ample opportunities and rewards for historians and anthropologists to engage in virus hunting in fieldnotes and archives alike. Taking outbreak narratives ethnographically seriously is essential if we are to resist this temptation and its neocolonial agenda of epistemological enclosure and extraction. Virus hunting as a historical or anthropological practice reduces and denies the autonomy of indigenous ways of knowing and being. At the same time, as demonstrated in this article, it also obscures the political agendas that, more often than not, directly affect the ontological and epistemological contours of colonial and hegemonic outbreak narratives.

## Footnotes

1 Albert Camus, *The Plague*, trans. Stuart Gilbert (Harmondsworth: Penguin, 1960). Originally published as *La peste* (Paris: Gallimard, 1947).

2 Camus, *The Plague*, 9.

3 Ibid., 15.

4 On rats in *The Plague*, including the symbolic and narrative function of their out-of-placedness see Macs Smith, “What Dies in the Street: Camus’s La Peste and Infected Networks,” *French Forum* 41 (2016): 193-208; Colin Davis, “Camus’s ‘La Peste’: Sanitation, Rats, and Messy Ethics,” *Modern Language Review* 102 (2007): 1008-1020; Charles E. Rosenberg, “What Is an Epidemic? AIDS in Historical Perspective,” *Daedalus* 118 (1989): 1-17; Elana Gomel, “The Plague of Utopias: Pestilence and the Apocalyptic Body,” *Twentieth Century Literature* 46 (2000): 405-433. For some examples on Camus and Covid see Elisabeth Stelson, “COVID and Camus: Reflections on *The Plague*, Collective Experience, and Qualitative Inquiry during a Pandemic,” *Qualitative Social Work* 20 (2021): 41–47; Robert Rickard, “Camus’s *The Plague*: A Guidebook for Covid-19,” *Royal Society of Arts*, 4 August 2021, https://www.thersa.org/comment/2021/08/camuss-the-plague; César Pérez Romero, “A Mirror in Fiction: Drawing Parallelisms between Camus’s *La Peste* and COVID-19,” *BMJ Medical Humanities* 47 (2021): e4; Robert Zaretsky, “Out of a Clear Blue Sky: Camus’s *The Plague* and Coronavirus,” *Times Literary Supplement*, 10 April 2020, https://www.the-tls.co.uk/articles/albert-camus-the-plague-coronavirus-essay-robert-zaretsky/.

5 Myron Echenberg, *Plague Ports: The Global Urban Impact of Bubonic Plague 1894-1901* (New York: New York University Press, 2007).

6 In understanding figures as “repetitive tropes, phrases, images, or ideas that shape our encounters with the world,” I follow Laura A. Ogden, *Loss and Wonder at the End of the World* (Durham: Duke University Press, 2021), 2.

7 Carol A. Benedict, *Bubonic Plague in Nineteenth-Century China* (Berkeley: Stanford University Press, 1996).

8 I am borrowing the term “outbreak narrative” from Priscilla Wald, *Contagious: Cultures, Carriers, and the Outbreak Narrative* (Durham: Duke University Press, 2008).

9 For an extensive discussion of how this approach formed part of the emergence of modern epidemiology, see Christos Lynteris, *Ethnographic Plague: Configuring Diseases on the Chinese-Russian Frontier* (London: Palgrave Macmillan, 2016).

10 Ibid.; Christos Lynteris, “Mahamari Plague: Rats, Colonial Medicine and Indigenous Knowledge in Kumaon and Garhwal, India,” *Medical Anthropology* 41 (2022): 373-386.

11 Eduardo Viveiros de Castro, *Le regard du jaguar. Introduction au perspectivisme amérindien*, trans. Pierre Delgado (Bordeaux: Éditions la tempête, 2021), 33.

12 See for example the debate on whether the 1889 flu pandemic was really caused by a coronavirus. Patrick Berche, “The Enigma of the 1889 Russian Flu Pandemic: A Coronavirus?” *La presse médicale* 51 (2022): 104111; Gina Kolata, “An Undiscovered Coronavirus? The Mystery of the ‘Russian Flu,’” *New York Times*, 14 February 2022, https://www.nytimes.com/2022/02/14/health/russian-flu-coronavirus.html.

13 Benedict, *Bubonic Plague in Nineteenth-Century China*; Florence Bretelle-Establet, “Les épidémies en Chine à la croisée des savoirs et des imaginaires: le Grand Sud aux xviii et xix siècles?” *Extrême-orient extrême-occident* 37 (2014): 21-60; Echenberg, *Plague Ports.*

14 Tamara Ben-Aria, Simon Neerinckx, Lydiane Agier, Bernard Cazelles, Lei Xu, Zhibin Zhang, Xiye Fang, Shuchun Wang, Qiyong Liu, Nils C. Stenseth, “Identification of Chinese Plague Foci from Long-Term Epidemiological Data,” *PNAS* 109 (2012): 8196-8201; Peng Wang, Liyuan Shi, Fuxin Zhang, Ying Guo, Zhikai Zhang, Hongli Tan, Zhigang Cui, Yibo Ding, Ying Liang, Yun Liang, Dongzheng Yu, Jianguo Xu, Wei Li, Zhizhong Song, “Ten Years of Surveillance of the Yulong Plague Focus in China and the Molecular Typing and Source Tracing of the Isolates,” *PLoS Neglected Tropical Diseases* 12 (2018): e0006352.

15 Benedict, *Bubonic Plague in Nineteenth-Century China*; Wilt L. Idema, “The Precious Scroll of the Rat Epidemic by Li Shanbao,” *Sino-Platonic Papers* 313 (2021), https://sino-platonic.org/complete/spp313_Precious_Scroll_Rat_Epidemic.pdf. See also Di Lu, “History of Epidemics in China: Some Reflections on the Role of Animals,” *Asian Medicine* 16 (2021): 137-152.

16 Lynteris, “Mahamari Plague.” The first systematic comparison between ratfalls in the Indian Himalayas and in Yunnan appears in 1878. House of Commons Parliamentary Papers (HCPP), C.2130-I, Memorandum by Mr. J. Netter Radcliffe on the Progress of Levantine Plague in 1877 and part of 1878; together with an Account of an Outbreak of the Bubonic Plague in India (Mahamari) in 1876-1877, 139-143. French medical experts were, however, aware of Mahamari and its connection to ratfalls before the proliferation of the Yunnan plague in missionary or medical writings. Adrien Proust, *Essai sur l’hygiène internationale; ses applications contre la peste, la fièvre jaune et le choléra asiatique* (Paris: G. Masson, 1873), esp. 93-94. The Francophone general public was exposed to the phenomenon as early as 1862 through a sensationalist account. Jules Gérard Jules Gérard*, Voyages et chasses dans l’Himalaya* (Paris: M. Lévy frères, 1862).

17 Neil Pemberton, “The Rat-Catcher’s Prank: Interspecies Cunningness and Scavenging in Henry Mayhew’s London,” *Journal of Victorian Culture* 19 (2014): 520–535.

18 Archives Œuvres Pontificales Missionnaires (Lyon), I-7, Séminaire des Missions Étrangères de Paris 1863-1894, No. 71-242.

19 David G. Atwill, *The Chinese Sultanate: Islam, Ethnicity, and the Panthay Rebellion in Southwest China, 1856-1873* (Berkeley: Stanford University Press, 2006).

20 Ji Li, ed., *Missions Étrangères de Paris (MEP) and China from the Seventeenth Century to the Present* (Leiden: Brill, 2021).

21 Institut de recherche France-Asie (IRFA) Archives, AMEP 542, Ponsot à MM. les membres des Conseils centraux de l’Œuvre de la Propagation de la Foi, 27 July 1874, 252-256.

22 Ibid. The term that Ponsot attributes to Chinese framings of the disease at the time is *Cang-tsy-tchén* (later transcribed as Yang-tsy-tchén, *Yang-tse-tchén* and *Yang-tzŭ*; etymology discussed below) and not *shuyi*, which would be the standard Chinese term for a rat disease and which was later used in Chinese medical works to mean bubonic plague. With the exception of “Yunnan,” in this article I have maintained location spellings as they appear in the sources.

23 Vicariat Apostolique du Yun-Nan, “Lettre de Mgr Ponsot, vicaire apostolique du Yun-nan, à MM. les membres des Conseils centraux de l’Œuvre de la Propagation de la Foi, Yun-nan, 27 juillet 1874,” *Annales de la propagation de la foi* XLVII (1875): 242-250, 247-248. The Le Guilcher/Ponsot narrative was later reproduced in an oft-quoted Catholic publication with wide distribution. “Les épidémies au Yun-nan,” *Les missions catholiques: Bulletin hebdomadaire de L’Œuvre de la Propagation de la Foi* 911 (1886): 555-557. Father Tchao was said to have returned to Pien-kio and to have contracted and succumbed to the disease. Ibid., 248. However, another report has him as dying of cholera. “Nécrologie,” *Les missions catholiques: Bulletin hebdomadaire de L’Œuvre de la Propagation de la Foi* 7 (1875): 34. Ponsot also later claimed that the epidemic was one of cholera rather than plague. “Lettre de Mgr Ponsot… à M. le Directeur général de l’ Œuvres de la Sainte-Enfance, Yun-nan, 20 octobre 1880,” *Annales de l’Œuvres de la Sainte-Enfance* 32 (1881): 171-176.

24 Fleeing to the hills was not always a spontaneous response to plague. Jean-Antoine-Louis Terrasse, who was killed and allegedly eaten in a massacre near Dali in 1883, was said to have gathered the converted Pien-Kio inhabitants upon the first signs of the disease and to have evacuated them to the hills where they remained unaffected by it. “Les épidémies au Yun-nan.”

25 The notion that plague spread from rats to humans was not part of British colonial medical approaches to Mahamari up until the 1870s, with rats being seen as simply co-patients of the disease. Lynteris, “Mahamari Plague.”

26 See for example IRFA Archives, AMEP 542, 419-421, Mandrat à Dubernard, 2 December 1877. The apostolic summary for 1874 noted Ponsot’s report on the epidemic, but made no mention of rats. IRFA Library, Le Séminaire de Missions Étrangères de Paris, Lettre Commune No.2, Paris, 31 décembre 1874.

27 S.T. Wang, *The Margary Affair and the Chefoo Agreement* (Oxford: Oxford University Press, 1940). In his diary of the expedition, Grosvenor made no mention of Fenouil’s plague narrative, instead describing the disease encountered by on 29-30 April 1876 as malaria. National Archives (UK), FO 17/747, Yunnan Mission, murder of Mr Margary, Mr Grosvenor’s Diary, Volume 6.

28 HCPP, C.1994, China No. 3 (1878), *Report by Mr. Baber on the Route Followed by Mr Grosvenor’s Mission Between Tali-Gu and Momein* (London: Harrison and Sons, 1878).

29 Ibid., 23.

30 Ibid. From across the voluminous MEP archives in Paris and Lyon surviving from his troubled time as provicar and then bishop in Yunnan, the only mention of the disease by Fenouil is the following: “Plague makes every year the most terrifying ravages. Two years in a row we saw a quarter of what remained of the population die, both Christians and pagans. New conversions cannot suffice to fill so many gaps.” IRFA Archives, 0545, Fenouil, Jean Joseph (correspondence), Fenouil à à MM. les membres des Conseils centraux de l’Œuvre de la Propagation de la Foi, 5 October 1882. In 1894, Fenouil provided an account of the 1870s epidemic that repeated his testimony to Baber without significant changes. “Dr. J. K. Michoud’s Report on the Health of Mengsz for the year ended 30^th^ April 1894,” China Imperial Maritime Customs II Special Series: No. 2 Medical Reports for the Year Ended 30^th^ September 1894, 47^th^ and 48^th^ Issues (1896): 32-56.

31 Patrick Manson, “Dr. Manson’s Report on the Health of Amoy for the Half-year ended 31^st^ March 1878,” *Imperial Maritime Customs* II. Special Series: No. 2. Medical Reports for the Half-Year Ended 31^st^ March, 1878, 15^th^ Issue, 25-26.

32 Marta Hanson, “Visualizing the Geography of the Diseases of China: Western Disease Maps from Analytical Tools to Tools of Empire, Sovereignty, and Public Health Propaganda, 1878–1929,” *Science in Context* 30 (2017): 219-280, 231. For an example of the term being interpreted in terms of boils see Joseph Ponsot, “Lettre à MM. les membres des Conseils centraux de l’Œuvre de la Propagation de la Foi (3 sept. 1877),” *Annales de la propagation de la foi* L (1878): 238-253, 240; Alexander Rennie, “Report on the Plague Prevailing in Canton during the Spring and Summer of 1894,” *China Imperial Maritime Customs* II Special Series No. 2 Medical Reports for the year ended 30^th^ Sep. 1894, 47^th^ and 48^th^ issues (1895): 65-72.

33 Manson, “Dr. Manson’s Report on the Health of Amoy,” 26.

34 Ibid.

35 Rocher, *La province chinoise du Yün-nan*, II, 279.

36 Jean Michaud, *“Incidental” Ethnographers: French Catholic Missions on the Tonkin-Yunnan Frontier, 1880–1930* (Leiden: Brill 2007), 100. Dupuis’s ultimate aim was to open Yunnan to French trade and influence by establishing a route via the Red River to Tonkin, an area in which annexation by the French Empire he would play a pivotal role.

37 Ibid.

38 Ibid.

39 Benedict, *Bubonic Plague in Nineteenth-Century China*; Bretelle-Establet, “Les épidémies en Chine à la croisée des savoirs et des imaginaires”; Anne Hardy, “Animals, Disease, and Man: Making Connections,” *Perspectives in Biology and Medicine* 46 (2003): 200-215; Barbara Bramanti, Katharine R. Dean, Lars Walløe, Nils Chr. Stenseth, “The Third Plague Pandemic in Europe,” *Proceedings of the Royal Society B* 286 (2019): 20182429.

40 Rocher, *La province chinoise du Yün-nan*, I, 75.

41 Ibid. The “buttons of Aleppo” are in fact a symptom of cutaneous leishmaniasis.

42 Baber’s text may have been available in translation to Rocher, as it had been published in 1878 in the *Bulletin de la Société de Géographie d’Anvers,* with no significant differences in the evocative passage describing ratfalls and the death of other animals. The translation was read at the 16 September 1878 meeting of the Geographic Society of Antwerp. Louis Delgeur, “Compte-rendu du rapport de M. C. E. Baber sur la route entre Tali-fou et Momein suivie par la mission de M. Grosvenor,” *Bulletin de la Société de Géographie d’Anvers* 3 (1878): 75-98.

43 Rocher, *La province chinoise du Yün-nan*, I, 211.

44 “French Land Trade with Southern China,” *The Times* 33119 (17 September 1890): 10.

45 See for example HCPP, C.5371, China No. 1 (1888). Report by Mr. F.S.A. Bourne of a journey in south-western China, 25.

46 HCPP, C.1994, 23.

47 Manson, “Dr. Manson’s Report on the Health of Amoy,” 26; Rocher, *La province chinoise du Yün-nan*, I, 75.

48 G.3.478–85, trans. in Hunter H. Gardner, *Pestilence and the Body in Latin Literature* (Oxford: Oxford University Press, 2019), 121.

49 Gardner, *Pestilence and the Body in Latin Literature*, 115.

50 Ibid.

51 I.F.C. Hecker, *The Black Death in the Fourteenth Century*, trans. B.G. Babington (London: A. Schloss, 1833 [1832]); Nükhet Varlık, “Why is Black Death Black? European Gothic Imaginaries of ‘Oriental’ Plague,” in *Plague Image and Imagination from Medieval to Modern Times*, ed. Christos Lynteris (London: Palgrave Macmillan, 2021), 11-35, 20.

52 Hecker, *Black Death in the Fourteenth Century,* 12.

53 Faye Marie Getz, “Black Death and the Silver Lining: Meaning, Continuity, and Revolutionary Change in Histories of Medieval Plague,” *Journal of the History of Biology* 24 (1991): 265-289.

54 Ibid., 276.

55 M 7.536–51 in Gardner, *Pestilence and the Body in Latin Literature*, 156.

56 This potential is evident in a two-part article in *Les missions catholic*, the bulletin of the Propagation de la Foi, published in 1886, which provides an extensive discussion of epidemics in Yunnan. “Les épidémies au Yun-nan,” 556. Li Guoqiang has also noted the proliferation of “miraculous” interventions on the part of MEP missionaries against plague in this context. Li Guoqiang, “Dapingzi, la première chrétienté au cœur du Yunnan (1835-1925),” *Bulletin de l’École française d’Extrême-Orient* 106 (2020): 267-320.

57 “Les épidémies au Yun-nan,” 556.

58
https://www.gutenberg.org/files/23700/23700-h/23700-h.htm. On Boccaccio’s approach to animals see Cormac Ó. Cuilleanáin, “Man and Beast in the ‘Decameron,’” *Modern Language Review* 75 (1980): 86-93. It is possible that Boccaccio’s hog image was in turn a loan from a description of mice and other animals fleeing their nests and wondering as if intoxicated in the open in Ibn Sina’s (Avicenna) tenth-century description of plague outbreaks. However, in the latter mice and rats do not die of the disease. “Plague and Fleas,” *Nature* 1986 (1907): 59-60. For a discussion of this as a variation on classical tropes of epidemic-related “inversion of nature” and the reception of the image in medieval and early modern plague literature, see Samuel K. Cohn, Jr, “Epidemiology of the Black Death and Successive Waves of Plague,” *Medical History Supplement* 27 (2008): 74-100, 78.

59 Hecker, *Black Death in the Fourteenth Century,* 12.

60 Gardner, *Pestilence and the Body in Latin Literature.*

61 David West, “Two Plagues: Virgil, Georgics 3.478–566 and Lucretius 6.1090–1286,” in *Creative Imitation and Latin Literature*, ed. David West and Rony Woodman (Cambridge: Cambridge University Press, 1979), 85.

62 Gardner, *Pestilence and the Body in Latin Literature*, 127. It is perhaps not accidental that we find a similar image of “tranquillity” following the discordant effects of plague in Rocher’s image of a “suspended life.” “No domestic animals, no more smoke rising from the chimneys, no noise, no movement; life seemed suspended.” Rocher, *La province chinoise du Yün-nan*, I, 211.

63 Matthew Crofts and Janine Hatter, “‘Rats Is Bogies I Tell You, and Bogies Is Rats’: Rats, Repression, and the Gothic Mode,” in *Gothic Animals: Uncanny Otherness and the Animal With-Out,* ed. Ruth Heholt and Melissa Edmunson (London: Palgrave Macmillan, 2020), 127-140.

64 Rachel Bruzzone, “*Polemos*, *Pathemata*, and Plague: Thucydides’ Narrative and the Tradition of Upheaval,” *Greek, Roman, and Byzantine Studies* 57 (2017): 882-909, 889, 894.

